# PIEZO1 promotes psoriasis-like skin inflammation in mice via NF-κB/IL-17 signaling pathway activation

**DOI:** 10.1186/s10020-025-01279-2

**Published:** 2025-06-10

**Authors:** Wen Li, Kan Ze, Xufeng He, Lili Yang, Huimin Zhang, Weian Yuan, Wuqing Wang

**Affiliations:** 1 Department of Dermatology, Shuguang Hospital Affiliated to Shanghai University of TCM, 528 Zhangheng Road, Pudong New Area, Shanghai, 201203 China; 2 Clinical Research Center, Shuguang Hospital Affiliated to Shanghai University of TCM, 528 Zhangheng Road, Pudong New Area, Shanghai, 201203 China; 3 Derpartment of Surgery VIII (Dermatology and Sores), Shanghai Municipal Hospital of Traditional Chinese Medicine, Shanghai University of Traditional Chinese Medicine, Shanghai, 200071 China; 4 Department of Dermatology, Minhang Hospital, Fudan University, Central Hospital of Minhang District, 170 Xinsong Road, Xinzhuang Town, Minhang District, Shanghai, 201100 China

**Keywords:** PIEZO1, Psoriasis, IL-17, NF-kB, Inflammation, Keratinocyte, Th17 cells

## Abstract

**Background:**

Psoriasis is a chronic inflammatory skin disorder characterized by hyperproliferative keratinocytes and an altered immune response. *PIEZO1*, a mechanically activated ion channel, has been implicated in various cellular processes, but its role in psoriasis pathogenesis remains unclear.

**Methods:**

We examined *PIEZO1* expression in skin samples from psoriatic patients and healthy individuals using western blot, immunohistochemistry, and mRNA expression analyses. Subsequently, we employed *PIEZO1* knock-out (KO) mice to establish imiquimod (IMQ)-induced psoriasiform models for in vivo experiments. Additionally, we conducted in vitro experiments with *PIEZO1*-silenced human keratinocytes (HaCaT cells) to investigate the impact on keratinocyte function and the expression of inflammatory cytokines.

**Results:**

*PIEZO1* expression was significantly upregulated in the basal layer of psoriatic lesions compared to healthy controls. In vivo, *PIEZO1* KO mice showed attenuated psoriasis-like symptoms, reduced keratinocyte proliferation, inflammatory cell infiltration, and less Th17 cells compared to wild-type mice. Loss of *PIEZO1* in vitro inhibited keratinocyte proliferation and migration, while inducing apoptosis. Transcriptome sequencing and subsequent analyses revealed that *PIEZO1* knockdown modulates the NF-kB signaling pathway and associated inflammatory genes. The in vitro activation of NF-kB signaling was diminished by *PIEZO1* silencing in keratinocytes, resulting in decreased inflammatory cytokine and chemokine expression. Furthermore, *PIEZO1* facilitated keratinocyte-mediated CD4 + T cell differentiation into Th17 cells, a key pathogenic factor in psoriasis.

**Conclusion:**

This study highlights the critical role of *PIEZO1* in psoriatic skin inflammation and suggests that *PIEZO1* may serve as a novel therapeutic target for psoriasis treatment. Our findings reveal that *PIEZO1* modulates keratinocyte proliferation, immune cell infiltration, and T cell differentiation through the NF-kB signaling pathway, contributing to the complex pathophysiology of psoriasis.

**Graphical Abstract:**

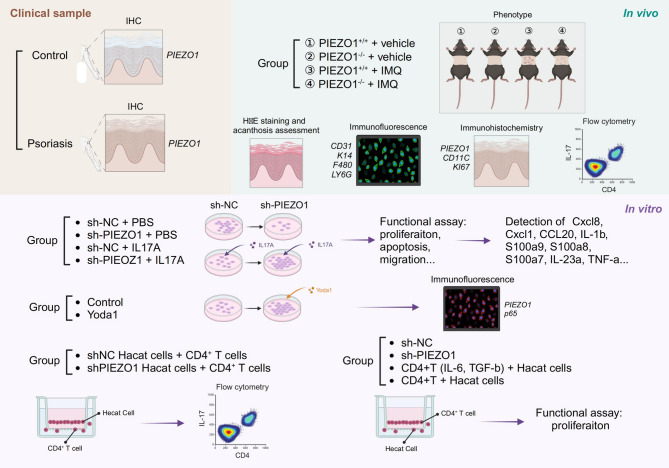

## Introduction

Psoriasis is a chronic, inflammatory, and systemic immune-mediated condition, presenting with distinctive erythematous plaques and squamous lesions (Campanati et al. 2021). Worldwide epidemiological evidence indicates a variable prevalence of psoriasis, with approximately 2–3% of the population affected, irrespective of age (Parisi et al. 2020). The disease is marked by heightened keratinocyte proliferation, atypical differentiation, and a substantial inflammatory milieu orchestrated by multiple immune cells, including T cells and dendritic cells (Chieosilapatham et al. 2021). Although the exact etiology of psoriasis is elusive, its pathogenesis is recognized to involve a multifaceted interplay of genetic predispositions, environmental factors, and immune responses.

Psoriasis exemplifies an immune cell-mediated skin disorder with keratinocyte dysfunction and perturbations in both innate and adaptive immunity, especially mechanisms involving T cells (Li et al. 2023). The cytokine interleukin-23 (IL-23), crucial in early inflammatory pathways in psoriasis(Li et al. 2024), prompts T cell activation. Consequently, activated T cells secrete a suite of inflammatory cytokines, notably IL-17 A, IL-17 F, IL-22, IL-6, and tumor necrosis factor-alpha (TNF-α) (Mills 2023). IL-23 is particularly instrumental in psoriasis for maintaining the cytokine-secreting phenotype of pathogenic T helper 17 (Th17) cells (Jacobse et al. 2023). The critical pathogenic role of Th17 cells and IL-17 A is underscored by the marked therapeutic efficacy of IL-17 A inhibitors in managing moderate-to-severe plaque psoriasis (He et al. 2024). Additionally, the nuclear transcription factor NF-κB interlinks altered states of keratinocytes and immune cells, influencing cellular proliferation, differentiation, apoptosis, and the production of cytokines and chemokines (Kumari et al. 2021). Among proinflammatory cytokines, TNF-α is prominent in the pathogenesis of psoriasis (Li et al. 2019). Following stimulation by TNF-α, the NF-κB p65 subunit relocates to the nucleus, enhancing the transcription of inflammatory mediators. TNF-α also elevates the synthesis of other cytokines such as interferon-γ (IFN-γ), IL-1β, and IL-6 by activated T lymphocytes and keratinocytes, contributing to inflammation and the psoriatic pathogenesis (Tonel and Conrad 2009). Nevertheless, the intricate molecular mechanisms linking keratinocyte dysfunction and subsequent immune activation are not fully elucidated.

The identification of molecular entities that govern psoriatic pathology is critical for innovating therapeutic strategies. *PIEZO1* is implicated in various cellular processes, such as mechanotransduction, cellular proliferation, and migration, and is now recognized for its role in inflammation and immunomodulation (Atcha et al. 2021). There is accumulating evidence for mechanosensitive ion channels’ involvement in critical cellular functions, including proliferation, migration, and apoptosis, pertinent to inflammatory skin diseases (Zhu et al. 2023; Xia et al. 2024). However, the specific contributions of *PIEZO1* to the pathophysiology of psoriasis remain to be clarified. Understanding the molecular effects of *PIEZO1* on keratinocyte action and immune signaling is imperative for devising targeted psoriasis therapies.

In this investigation, we evaluate the role of *PIEZO1* in psoriatic pathology by probing its expression in the epidermal basal layer of both human psoriatic lesions and an imiquimod (IMQ)-induced psoriasiform mouse model. We delve into the ramifications of *PIEZO1* absence, particularly regarding keratinocyte proliferation, apoptosis, migration, and the modulation of NF-κB signaling, a pivotal regulator of proinflammatory mediators and chemokines. Our findings will elucidate the molecular nexus between *PIEZO1* activation, NF-κB signaling in keratinocytes, and the ensuant inflammatory response along with Th17 cell differentiation pivotal to psoriatic pathology. *PIEZO1* incitement initiates pronounced NF-κB pathway activation, fostering increased secretion of chemokines, including CCL20 and CXCL1, attracting and activating Th17 cells. When co-cultured with *PIEZO1*-activated keratinocytes, these Th17 cells display augmented differentiation and IL-17 production. Conversely, *PIEZO1* inhibition or knockdown diminishes NF-κB activation, dampens chemokine secretion, and curtails Th17 cell differentiation, highlighting *PIEZO1* as a prospective therapeutic target in psoriasis.

## Methods and materials

### Ethics statement

All experiments involving human samples were approved by the Institutional Review Board of Shuguang Hospital Affiliated to Shanghai University of TCM. Informed consent was obtained from all human participants. Animal experiments were conducted in accordance with the guidelines of the Institutional Animal Care and Use Committee of Shuguang Hospital Affiliated to Shanghai University of TCM. IRB approval number: 2021-954-29-01; IACUC approval number: SYXK (Shanghai) 2020-0009.

### Clinical sample collection

Lesional skin tissues were collected from psoriasis patients undergoing routine biopsies, and normal skin tissues were obtained from a healthy control group undergoing plastic surgery. All samples were anonymized.

### Animal model

Male C57BL/6 mice (8 weeks old, weighing 22 ± 2 g) were supplied by the Shanghai Jiesijie Laboratory Animals Co., Ltd (Shanghai, China). The animals were raised at the Shanghai Skin Disease Hospital’s Laboratory Animal Center, where they were housed in specified pathogen-free animal (SPF) grade cages under aseptic conditions at a standard temperature (23 ± 2 ℃), with a standard meal and water available at all times. An IMQ-induced psoriasis mouse model was used to mimic human psoriasis, in which 62.5 mg of IMQ at a concentration of 5% (trade name Aldara^®^, containing 50 mg IMQ per gram) was administered daily. Both wild-type (*n* = 6) and PIEZO1 knockout (PIEZO1-/-, *n* = 6) mice were treated topically with IMQ cream once daily for a total of 5 days. Control animals were treated with vehicle cream. *PIEZO1*-/- (KO) mice were generated using the CRISPR-Cas9 system. Correct gene targeting was confirmed by genotyping PCR and sequencing. Skin biopsies were collected for further analysis, and psoriasis severity was evaluated by psoriasis area and severity index (PASI) scoring. According to the clinical PASI scoring criteria, the PASI scoring was applied to assess the degree of the mouse skin’s condition. Three independent researchers were assigned to record the scores of 0–4 (0, none; 1, slight; 2, moderate; 3, severe; and 4, extremely severe) for scaling, erythema, and thickness. The overall score (The sum of erythema, scales, and thickness scores) on a scale of 0–12 was applied to objectively quantify the severity of the psoriasis. The Acanthosis score was calculated on the basis of the thickness of the spinous layer and the number of cell layers on the following scale: score 0: normal, normal spinous layer thickness and normal number of cell layers. Score 1: slight thickening, slight thickening of the dull layer and slight increase in the number of cell layers. Score 2: moderate thickening, significant thickening of the dull layer and a significant increase in the number of cell layers. Score 3: severe thickening, extreme thickening of the dull layer and a significant increase in the number of cell layers.

### Cell culture

Human keratinocyte HaCaT cells were maintained in Dulbecco’s Modified Eagle’s Medium supplemented with 10% fetal bovine serum, 100 U/ml penicillin, and 100 µg/ml streptomycin at 37 °C in a humidified atmosphere containing 5% CO_2_.

### SiRNA transfection

For the knockdown experiments, HaCaT cells were transfected with two different siRNA constructs targeting PIEZO1 (siPIEZO1-1, siPIEZO1-2) or nontargeting control siRNA (si-NC) via the Lipofectamine RNAiMAX reagent (Invitrogen). The sequence of Piezo1-1 (targeting exon 23) is as follows: sense strand: 5’-GCAUGAAGCUCAACGUUAUTT-3’; antisense strand: 5’-AUAACGUUUGAGCUUCATGCTT-3’. The sequence of Piezo1-2 (targeting exon 15) is as follows: sense strand: 5’-CCUGGUCAUCUCATGCTT-3’. CCUGGUCAUCUUCGACAAATT-3’; antisense strand: 5’-UUUGUCGAAGAUGACCAGGTT-3’. The total amount of siRNA used was 3.75 µ/well.

### Hematoxylin and Eosin (HE) staining

After being extracted from the dorsal lesions, the skin samples were embedded in paraffin and preserved in a 4% paraformaldehyde solution. The samples underwent a gradual dehydration process through increasing concentrations of alcohol − 70%, 80%, 90%, and 95% for durations of 2 h, 1.5 h, 1 h, and another hour respectively. This was followed by two periods of thirty minutes each in both anhydrous alcohol and xylene. The specimens were then infiltrated with paraffin in three 30-minute intervals. Subsequently, the solidified tissue was embedded using a heated paraffin embedding system (Leica, Germany) and sectioned into 5-µm slices with a Semi-Automated Rotary Microtome (Leica, Germany). The slices underwent a staining protocol that involved two 10-minute xylene baths, sequential alcohol dehydrations in decreasing concentrations for 3 min each, and washing with running water. HE staining followed this process, which included a series of quick dips in acid alcohol and a bluing reagent coupled with brief water rinses. Finally, the stained slices were dehydrated once again through a series of alcohols, cleared in xylene, and mounted with neutral resin. The skin tissues were examined under an Inverted Research Microscope (Nikon, Japan) for any pathological changes.

### Protein extraction and western blot analysis

Skin tissues was performed using RIPA buffer supplemented with protease inhibitors. The protein concentration was quantified using a BCA Protein Assay Kit (Thermo Fisher Scientific, USA), and total proteins were separated using 10% SDSPAGE gels. The separated proteins were transferred to PVDF membranes and blocked with 5% skim milk. After blocking, the membranes were washed three times with TBST and incubated with primary antibodies including PIEZO1 (Proteintech, 15939-1-AP, 500 ug/ml, 1:1000 diluted) and β-actin (Proteintech, 81115-1-RR, 4H1, 1000 µg/ml, 1:1000 diluted) at 4 °C overnight, followed by appropriate HRP-conjugated secondary antibodiess (Proteintech, SA00001-1, 0.2 mg/mL, 1:5000 diluted) for 1 h at room temperature. The protein expression was measured using an enhanced chemilumin escence detection system (Thermo Fisher Scientific), and the relative expression of each band was quantified by an Image J system (National Institutes of Health, Bethesda, MD, USA).

### RNA isolation and quantitative RT-PCR (qRT-PCR)

Total RNA was extracted from mouse skin tissues and HaCaT cells using TRIzol reagent. cDNA synthesis was conducted using a reverse transcription kit (TAKARA, Japan). qRT-PCR was performed with SYBR Green PCR Master Mix on a real-time PCR system, and gene expression levels were normalized to GAPDH. Primer sequences for each gene (PIEZO1, IL-17 A, IL-23 A, TNF-A, IL-1B, S100 A7, and S100 A) were designed and optimized for efficiency. The relative expression of PIEZO1 was calculated using the 2^−ΔΔCt^ method. Primer sequences were as follows: PIEZO1 forward primer, 5’-TCTTCCTTAGCCATTACTACCT-3’, PIEZO1 reverse primer, 5’-TACGCTCCATCTGTCTTTTC-3’. IL-17 A forward primer, 5’-TCCCTCTGTGATCTGGGAAG-3’, IL-17 A reverse primer, 5’-CTC GAC CCT GAA AGT GAA GG-3’. IL-23 A forward primer, 5’-TGG CAT CGA GAA ACT GTG AGA-3’, IL-23 A reverse primer, 5’-TCA GTT CGT ATT GGT AGT CCT GTT A-3’. TNF-A forward primer, 5’-GATCGGTCCCCAAAGGGATG-3’, TNF-A reverse primer, 5’-CCACTTGGTGGTTTGTGAGTG-3’. IL-1B forward primer, 5’-AATGCCACCTTTTGACAGTGATG-3’, reverse primer, 5’-AGCTTCTCCACAGCCACAAT-3’. S100 A7, forward primer, 5’-AGGGTGAGGGTGATCTGTCC-3’, reverse primer, 5’-TTACTCTGTCCTCAGCCCTCC-3’. S100 A9 forward primer, 5’-CTGGAACGCAACATAGAGACC-3’, reverse primer, 5’-CGCCATCAGCATGATGAACT3’. GAPDH forward primer, 5’-GGGAAACTGTGGCGTGAT-3’, GAPDH reverse primer, 5’-GAGTGGGTGTCGCTGTTGA-3’.

### Immunohistochemistry and immunofluorescence

Paraffin-embedded skin sections were deparaffinized, rehydrated, and subjected to antigen retrieval. For IHC, endogenous peroxidase was quenched and sections were blocked with serum before incubation with the primary antibody against PIEZO1. Visualization was accomplished using a DAB substrate kit, and sections were counterstained with hematoxylin. IHC was performed using antibodies against PIEZO1, Ki67, and CD11c (1:1000 diluted). For tissue immunofluorescence, sections were double-stained with CD31/K14 and single-stained with F480 and LYG6. Appropriate fluorescence-conjugated secondary antibodies were then applied. For cellular immunofluorescence, PIEZO1 and p65 were stained after HaCaT cells were treated with 20 µM Yoda1 for 1 h. Nuclear staining was achieved with DAPI, and images were captured using a fluorescence microscope. Semi-quantification of the image was conducted using Image-Pro Plus 6.0.

### 5-ethynyl-2’-deoxyuridine (EdU) incorporation assay

EdU incorporation experiments were performed on HaCaT cells via the Cell-Light EdU Apollo 488 In Vitro Imaging Kit (Beyotime Company, Shanghai, China) according to the manufacturer’s instructions. Images were acquired using an Olympus DP70 microscope (Olympus), and the enumeration of cells displaying positive EdU signals was performed.

### Annexin V/PI staining and flow cytometry

The quantification of apoptotic rates was performed using Annexin V-FITC/Propidium Iodide (PI) staining. Cells were harvested, suspended in binding buffer, and subjected to staining with Annexin V and PI (Tianjin Sungene Biotech Co., Tianjin, China). The fluorescence emitted by the cells was observed utilizing flow cytometry equipment (Sysmex Partec GmbH, Goerlitz, Germany).

### Transwell migration assay

The migratory ability of HaCaT cells was evaluated using a Transwell chamber assay (BD). Cell migration was assessed in 24-well Transwell chambers equipped with polycarbonate membranes containing pores measuring 8 μm. The upper chamber was seeded with transfected HaCaT cells, while the lower chamber contained a chemotactic stimulus consisting of DMEM supplemented with 10% FBS. Following incubation, non-migrating cells were removed and migrating cells were fixed at room temperature for 20 min using formaldehyde (Sinopharm Chemical Reagent Co.). Subsequently, the cells were rinsed with PBS and stained for 30 min using crystal violet solution (Solarbio). The number of cells present in each field of view was quantified under a microscope.

### CD4 + T cell isolation and co-culture experiments

CD4 + T cells were isolated from mouse spleen using the Dynal^®^ CD4 + negative isolation kit following the instructions provided by Thermo Fisher Scientific. The purified CD4 + T cells were stimulated in 24-well plates coated with anti-CD3 (Clone 145-2 C11, BioXcell) and anti-CD28 (Clone 37.51, BioXcell), at a density of 106 cells per well in RPMI media supplemented with Glutamax (RPMI Media 1640 1X + Glutamax, Gibco, Life Technologies, UK), FBS (Gibco, Life Technologies, Germany) at a concentration of 10%, L-glutamine at a concentration of 2 mM, penicillin at a concentration of 100 U/ml and streptomycin at a concentration of 100 µg/ml (Gibco, Life Technologies, USA). For coculture experiments, isolated T cells were combined with HaCaT cells previously transfected with sh-NC or shPiezo1 at 2 × 10^5^ cells per well. The co-cultures were conducted in the presence or absence of 30 ng/ml IL-6 and 5ng/ml TGF-β (PeproTech, USA) for a duration of 48 h using cell media. Following this, the harvested cells were added to activated T cells in T cell media on day 0 at a ratio of 1:25 (Hacat: T cell).

### RNA sequencing and bioinformatic analysis

Following the manufacturer’s guidelines, total RNA was extracted from dorsal skin tissues of mice using TRIzol reagent (Invitrogen, Carlsbad, CA, USA). The samples were then sent to Shanghai Biochip Co., Ltd. for RNA sequencing analysis on libraries prepared from skin tissues of both IMQ-treated WT and PIEZO1-/- mice. Data processing involved a bioinformatics pipeline that included gene expression quantification, alignment to the mouse reference genome and differential expression analysis. Only DEGs with fold change ≥ 1.5 and *P*-value < 0.05 (t-test) were selected for further analysis. GO and KEGG databases were used for functional annotation and pathway enrichment analyses respectively.

### Statistical analysis

The statistical analysis was performed using SPSS 23.0 software, and the results were presented as mean ± SD based on three independent experiments. For comparisons among three or more groups, a one-way ANOVA was employed. Prior to comparing two groups, normality testing was conducted; if the data exhibited a normal distribution, a t-test was utilized; for non-normally distributed data, a Kruskal-Wallis test was applied. Statistical significance was defined as *p*-values less than 0.05.

## Results

### Enhanced expression of *PIEZO1* in the basal layer of psoriatic skin

**Fig. 1:**
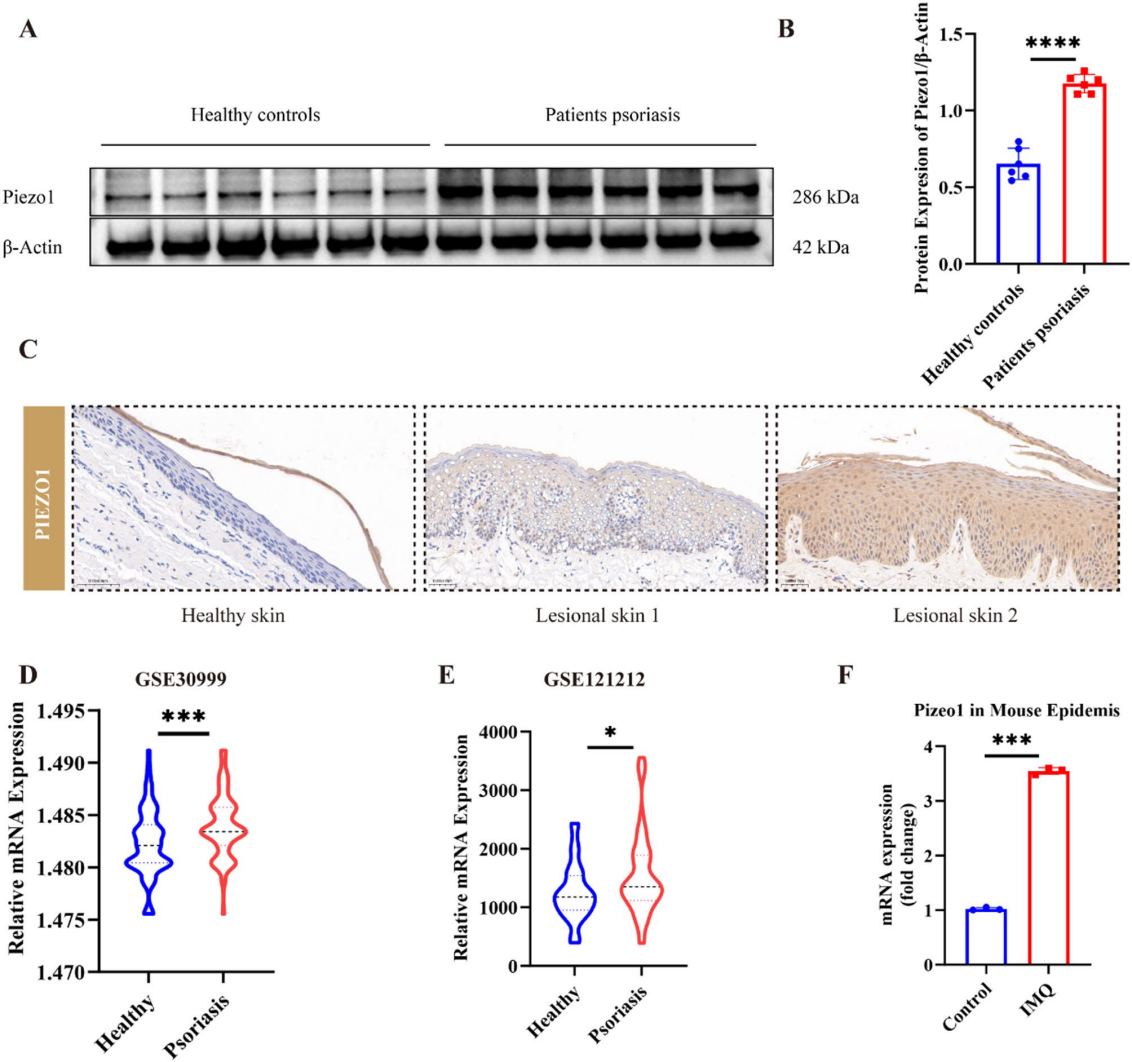
*PIEZO1* is highly expressed in patients with psoriasis. **A **Western blot analysis was performed to examine the protein expression of PIEZO1 in both psoriasis patients and the control group’s skin tissues. **B** Quantitative statistical analysis was conducted on the Western blot results. **C **Immunohistochemistry was employed to detect PIEZO1 protein expression in both psoriasis patients’ and controls’ skin tissues. **D** and **E **Public databases were utilized for analyzing PIEZO1 mRNA expression in both psoriasis patient’s and control’s skin tissues. (F) PIEZO1 mRNA expression in the skin tissue of the psoriasis-like IMQ mouse model and the control group. * *P*<0.05, ***P<0.001,**** *P*<0.0001

To elucidate the mechanistic role of *PIEZO1* in psoriasis, we harvested skin tissue samples from psoriatic patients and healthy individuals, processing them for subsequent analyses. WB results depicted in Fig. 1A and B demonstrate a significant upregulation of PIEZO1 protein in the basal layer of the epidermis in psoriatic lesions as opposed to its expression in healthy controls (*P* < 0.0001). IHC further corroborated these findings, as evidenced by the intensified PIEZO1 staining predominantly in the suprabasal layers of the psoriatic tissue, despite notable inter-patient heterogeneity (Fig. 1C). To complement these findings, we interrogated the expression profile of PIEZO1 at the mRNA level using publicly available datasets (GSE30999 and GSE121212). Bioinformatics analysis revealed a consistent elevation in PIEZO1 mRNA in lesional skin from psoriatic patients relative to controls (GSE30999: *P* < 0.001 and GSE121212: *P* < 0.05), as presented in Fig. 1D. Extending our investigation to an in vivo context, we employed an IMQ-induced mouse model resembling the human condition of psoriasis. This model exhibited a parallel increase in PIEZO1 mRNA expression compared to the untreated control group, validated through qRT-PCR as shown in Fig. 1F (*P* < 0.001). Collectively, these results indicate a prominent elevation in *PIEZO1* expression in the epidermal basal layer of psoriatic skin, suggesting its potential contribution to the pathophysiology of psoriasis.

### *PIEZO1* deficiency attenuates IMQ-induced psoriasiform phenotype in mice

**Fig. 2:**
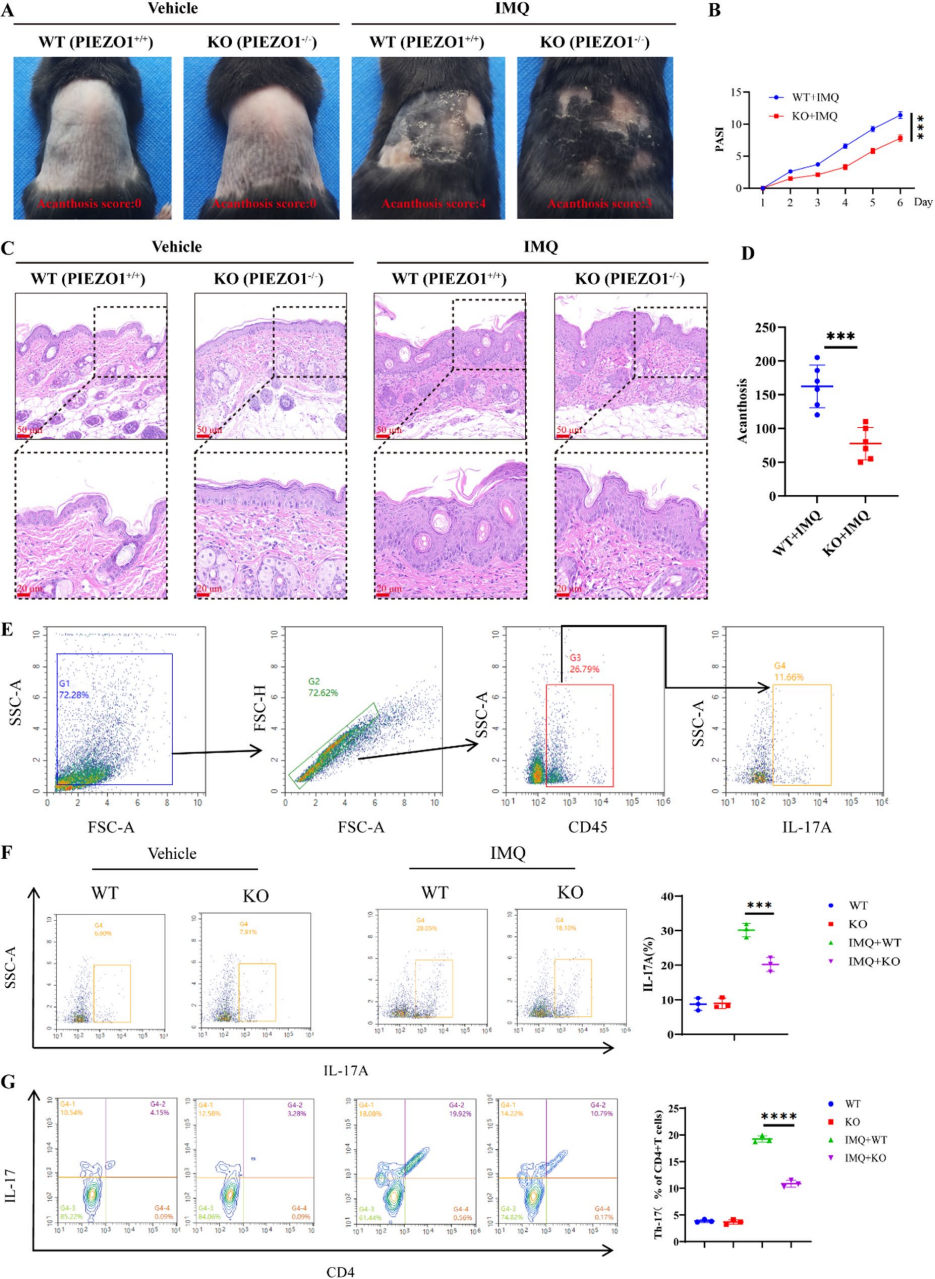
*PIEZO1* deficiency attenuates IMQ-induced psoriasiform phenotype in mice. **A **Skin lesions induced by IMQ were observed in both PIEZO1-/- mice (*n* = 6) and control mice (*n* = 6). **B **PASI score assessment was conducted. **C **HE staining was performed for histological analysis. **D **Acanthosis was evaluated. **E **Gates were set for flow analysis purposes. **F **Flow cytometric analysis determined the percentage of IL-17 A-producing cells. **G **The proportion of CD4 + T cells positive for IL-17 expression was analyzed using flow cytometry. ***P<0.001,**** *P*<0.0001

The role of *PIEZO1* in the development of psoriasis-like skin inflammation was elucidated using an IMQ-induced mouse model. WT mice, bearing intact *PIEZO1* alleles (*PIEZO1*+/+), upon IMQ application, displayed pronounced psoriasiform dermatitis, starkly contrasting the phenotype of untreated controls (Fig. 2A). Conversely, *PIEZO1* KO mice (*PIEZO1*-/-) showed a notably milder disease phenotype following IMQ treatment when compared to their WT counterparts. Quantitative assessment through PASI scoring indicated that IMQ-treated KO mice exhibited significantly lower scores than the WT + IMQ group, with the most pronounced difference evident on days 5 and 6 of treatment (Fig. 2B). Histopathological analysis through H&E staining of dermal tissues highlighted marked keratinocyte hyperproliferation and dense inflammatory cell infiltration in WT mice treated with IMQ, concurrent with a considerable increase in epidermal thickness. In stark contrast, KO mice receiving IMQ treatment exhibited notably fewer inflammatory cells, reduced keratinocyte proliferation, and diminished acanthosis and epidermal thickening (Fig. 2C). Following IMQ administration, the Acanthosis phenotypes in *PIEZO1* KO mice were significantly attenuated compared to those in the WT group (Fig. 2D). Quantitative analyses of Th17 immune cells in the dermal and splenic tissues of mice were performed via flow cytometry. After provoking the IMQ-induced psoriasiform model, there was a substantial increase in the proportion of Th17-positive cells in these tissues, as demonstrated in Figs. 2E–G. Importantly, *PIEZO1* KO mice subjected to IMQ treatment exhibited a notable reduction in this upsurge of Th17 cells compared to WT IMQ mice, suggesting that the absence of *PIEZO1* hinders Th17 cell proliferation and alleviates the emergence of psoriasis-like lesions.

### Deletion of *PIEZO1* hinders keratinocyte proliferation and alleviates inflammation

**Fig. 3:**
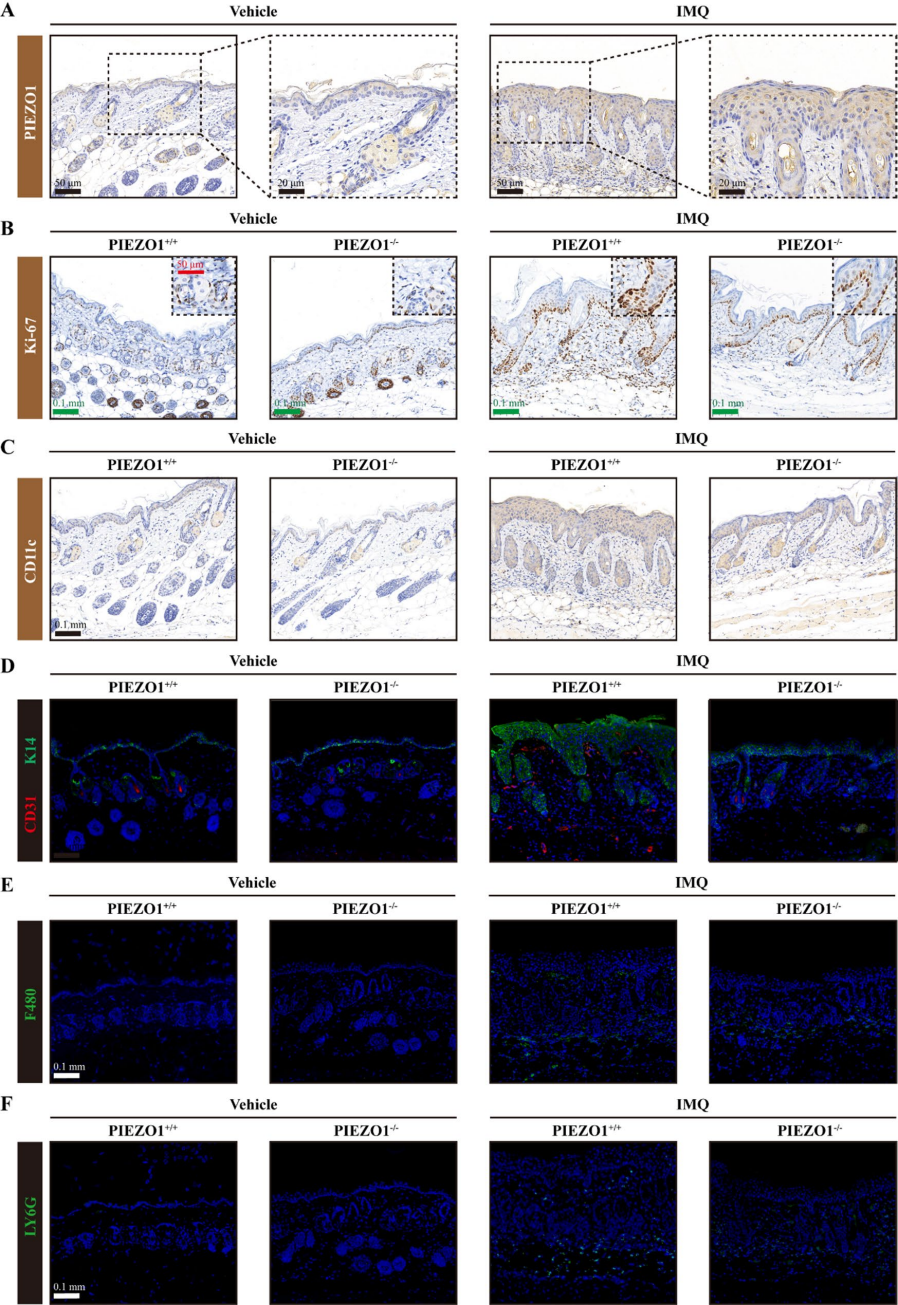
Deletion of *PIEZO1* impedes keratinocyte proliferation and mitigates inflammation in mouse skin tissue. **A **Immunohistochemical analysis of PIEZO1 expression in mouse skin tissue. **B **Immunohistochemical staining for Ki67 in mouse skin tissue. **C **Immunohistochemical detection of CD11 C in mouse skin tissue. **D **Dual immunofluorescent labeling of CD31 and K14 in mouse skin tissue. **E **Single immunofluorescent staining for F480 in mouse skin tissue. **F **Single immunofluorescent staining for LY6G in mouse skin tissue

In the IMQ-induced psoriasis mouse model, IHC analysis revealed an elevation in *PIEZO1* expression compared to control samples (Fig. 3A). *PIEZO1* staining intensity was markedly enhanced in the IMQ-treated wild-type (WT; PIEZO1+/+) group, signifying an upregulation of *PIEZO1* in response to IMQ application. Subsequent investigation into the Ki67 (a marker of cell proliferation) revealed a discernible augmentation in the WT group following IMQ treatment, pointing towards elevated keratinocyte proliferation (Fig. 3B). Contrastingly, this proliferative response was notably dampened in *PIEZO1-*deficient (KO; *PIEZO1-/-*) mice subjected to IMQ, highlighting the potential of PIEZO1 deletion in attenuating keratinocyte hyperproliferation. Investigating the inflammatory response, CD11c (a dendritic cell marker) expression was significantly escalated in IMQ-treated WT mice, indicative of heightened inflammation (Fig. 3C). This increase was substantially mitigated in *PIEZO1* KO mice, suggesting that *PIEZO1* deletion can reduce the inflammatory cell infiltrate. Through dual IF of *CD31* (an endothelial cell marker) and *keratin 14* (K14; a keratinocyte marker), we observed a pronounced increase in both markers in the WT mice receiving IMQ treatment, denoting an enhancement in keratinization and cell adhesion (Fig. 3D). The simultaneous decrease in the staining intensities for K14 and CD31 within the *PIEZO1* KO mice signifies a suppression in keratin production and cell-cell adhesion upon *PIEZO1* deletion. Further immunofluorescent assessment for *F4/80* (a macrophage marker) and *LY6G* (a neutrophil marker) indicated that IMQ administration resulted in the accentuation of these cells within the deep dermal layers of WT mice, compared to controls (Fig. 3E-F). Conversely, the intensities of both F4/80 and LY6G proteins were considerably reduced in the *PIEZO1* KO group, implicating the deletion of *PIEZO1* in the inhibition of macrophage and MDSC infiltration. Collectively, these findings substantiate the proposition that the ablation of *PIEZO1* mitigates the pathogenesis of psoriasis by reducing keratinocyte proliferation and curtailing the associated inflammatory response.

### Loss of *PIEZO1* suppresses proliferation and migration, and induces apoptosis in keratinocytes in vitro

**Fig. 4:**
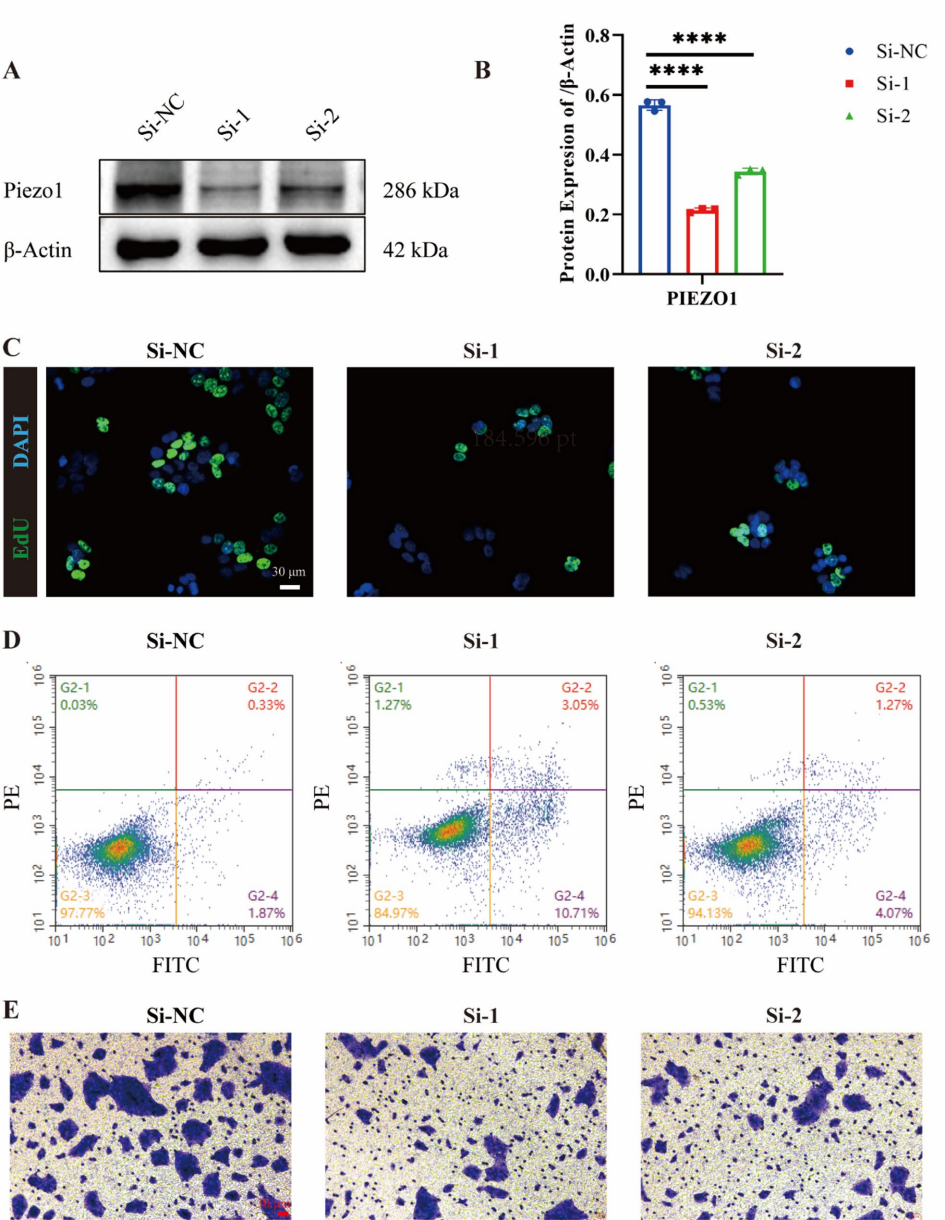
Suppression of *PIEZO1* leads to inhibition of proliferation and migration, as well as induction of apoptosis in vitro in keratinocytes. **A **& **B **Western blot analysis was performed to assess the knockdown efficiency of Piezo1 siRNA. **C **EdU assay was employed to evaluate cell proliferation following silencing of PIEZO1. **D **Annexin/V-PI staining flow cytometry analysis was utilized for detecting apoptotic cells after PIEZO1 silencing. **E** Transwell assay was used to measure the migratory capacity of cells upon silencing PIEZO1. **** *P*<0.0001

In Fig. 4A–B, the effectiveness of two siRNAs targeting *PIEZO1* (si-1 and si-2) was determined in human immortalized keratinocytes (HaCaT) compared to a negative control group transfected with non-targeting siRNA (NC). Efficient knockdown of *PIEZO1* was confirmed via WB analysis, which demonstrated a significant reduction in PIEZO1 protein levels in cells transfected with si-1 and si-2 (*P* < 0.001), with si-1 achieving higher knockdown efficiency than si-2. IF analysis provided additional confirmation of effective PIEZO1 knockdown. As shown in Fig. 4C, cells transfected with si-1 and si-2 displayed a significant reduction in EdU-labeled cells, indicating diminished proliferative capacity following PIEZO1 knockdown. Furthermore, the consequences of PIEZO1 loss on apoptosis were explored using Annexin V/PI staining followed by flow cytometry analysis. The data presented in Fig. 4D indicate that transfection with si-1 and si-2 markedly increased the apoptotic fraction in HaCaT cells compared to control, suggesting that PIEZO1 knockdown facilitates apoptosis. Cell migration, a critical aspect of keratinocyte biology, was assessed using a Transwell migration assay. Figure 4E shows that HaCaT cells with reduced *PIEZO1* expression due to si-1 or si-2 transfection exhibited significantly impaired migration capabilities. Collectively, these findings demonstrate that loss of *PIEZO1* expression in keratinocytes significantly attenuates proliferation and migration, while simultaneously promoting apoptosis, highlighting the crucial role of *PIEZO1* in the maintenance of keratinocyte homeostasis.

### *PIEZO1* modulates psoriatic pathogenesis via activation of the NF-kB signaling pathway

**Fig. 5:**
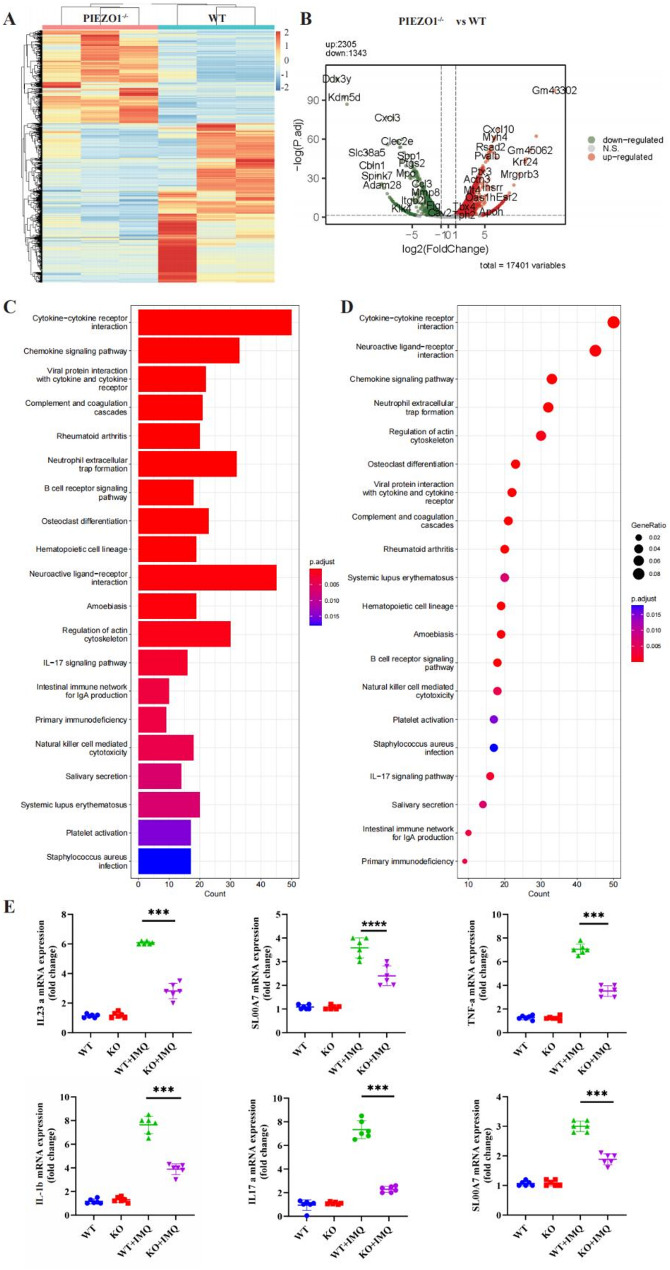
*PIEZO1* modulates psoriatic pathogenesis through activation of the NF-kB signaling pathway. **A **Heat map illustrating differentially expressed genes between WT and PIEZO1-/- mice. **B **Volcano plot depicting differentially expressed genes between WT and PIEZO1-/- mice. **C **GO pathway analysis revealing differential gene expression patterns. **D **KEGG pathway analysis highlighting differential gene expression profiles. **E **qPCR assessment of mRNA expression levels for inflammation-related genes. ***P<0.001,**** *P*<0.0001

To elucidate the role of *PIEZO1* in psoriatic pathogenesis we performed comprehensive transcriptome sequencing on skin tissues from both IMQ-induced WT mice and *PIEZO1* KO (*PIEZO1-/-*) counterparts. Differential gene expression analysis revealed a distinct pattern between WT and *PIEZO1-/-* groups, as depicted by the heatmap in Fig. 5A. The resultant gene expression signature emphasized the regulatory impact of *PIEZO1* on psoriatic skin. Subsequent analyses utilizing a volcano plot further clarified these expression differences, as exhibited in Fig. 5B. Notable upregulated genes in *PIEZO1-/-* mice included *Cxcl10* and *Ptx3*, whereas *Ptgs2* and *CCL3* were among the genes downregulated, suggesting a distinctive shift in inflammatory mediators upon *PIEZO1* disruption. GO and KEGG pathway enrichment analyses were conducted on differentially expressed genes to pinpoint functional categories and potential signaling cascades influenced by *PIEZO1*. Figure 5C and D present the enrichment results, implicating *PIEZO1* in the modulation of pathways such as cytokine-cytokine receptor interaction, chemokine signaling, and neuroactive ligand-receptor interaction. To validate the impact of *PIEZO1* on inflammatory signaling, we quantified the transcriptional levels of key inflammatory cytokines using qRT-PCR. As Fig. 5E delineates, IMQ treatment markedly elevated the mRNA expression of *IL-23a*,* S100 A7*,* TNF-α*,* IL-1β*, and *S100 A9* in mouse skin tissue. However, genetic ablation of *PIEZO1* considerably attenuated this upregulation, underscoring its inhibitory effect on the NF-kB signaling cascade during psoriatic inflammation. In summary, these data demonstrate the pivotal role of *PIEZO1* in psoriasis by modulating the transcription of genes associated with inflammatory responses, highlighting its function as a positive regulator of the NF-kB signaling pathway.

### *PIEZO1* enhances keratinocyte inflammation and chemokine expression by activating the *NF-kB* signaling pathway in vitro

**Fig. 6:**
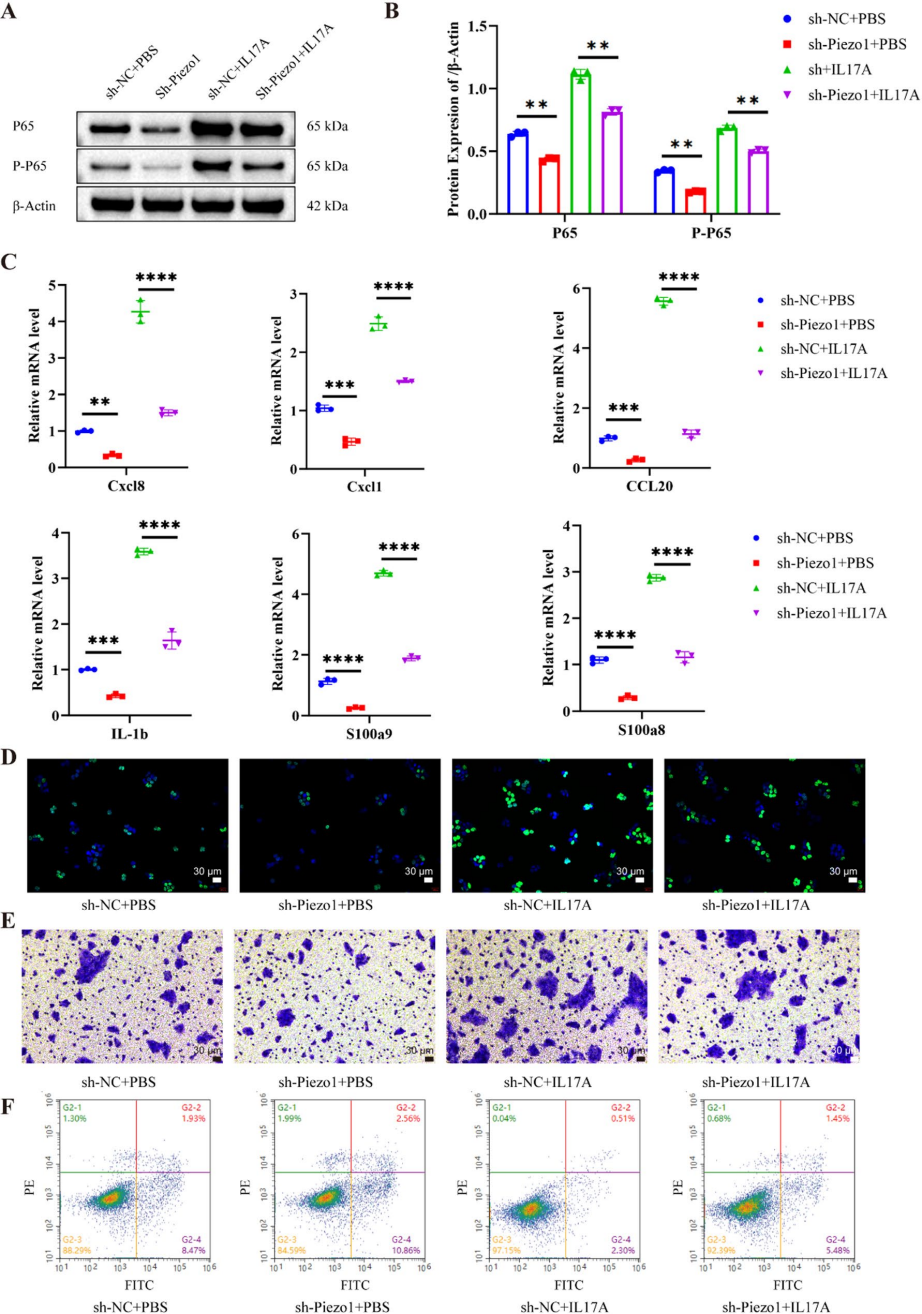
Enhanced activation of the NF-kB signaling pathway by *PIEZO1* promotes keratinocyte inflammation and upregulates chemokine expression in vitro. **A **Western blot detection of P65 and p-p65 protein expression levels **B** quantitative statistical analysis of Western blot **C **qPCR detection of inflammatory cytokine chemokine mRNA **D **EdU detection of cell proliferation **E **Transwell was used to detect cell migration ability **F **Annexin/V-PI was used to detect cell apoptosis ability. ***P<0.001,**** *P*<0.0001

**Fig. 7:**
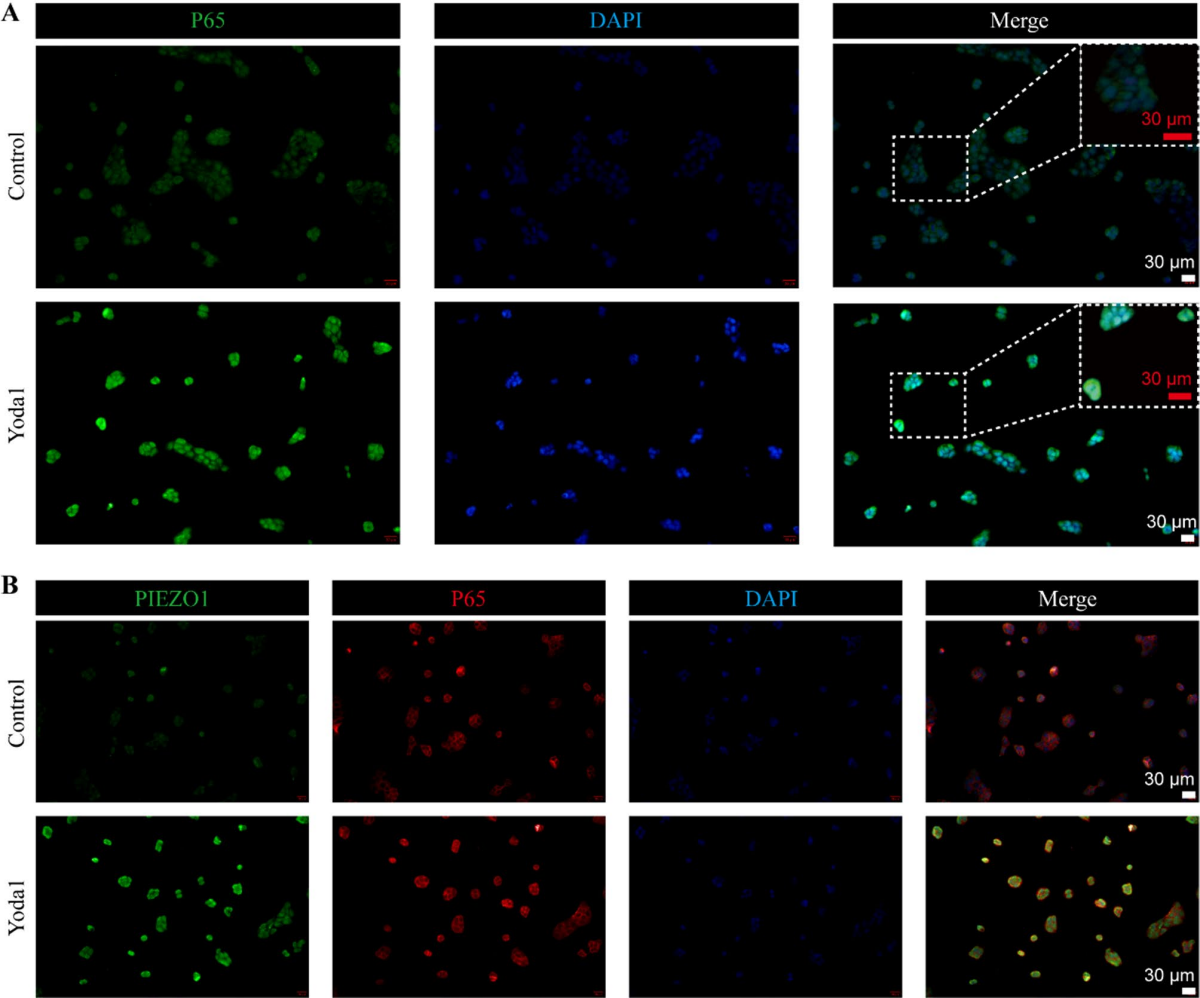
p65 was activated after *PIEZO1* upregulation by Yoda1. **A **Nuclear translocation detection of p65. **B **Immunofluorescence staining of double-stained PIEZO1 and p65

Figure 6A and B demonstrate a notable decrease in p65 protein levels, as evidenced by Western blot analysis, upon transfecting HaCaT cells with sh-Piezo1 compared to cells transfected with sh-NC. This is in contrast to the increased levels of phosphorylated p65 (p-p65) observed after treating the cells with 10 ng/ml of human recombinant IL-17 A. These results suggest that silencing Piezo1 attenuates p65 protein activity. Figure 6C shows a significant increase in mRNA expression of inflammatory markers, including IL-1β, S100a9, S100a8, and chemokines such as CXCL8, CXCL1, and CCL20 following IL-17 A treatment. This response is diminished when cells are transfected with sh-Piezo1 which suppresses these inflammatory mediators. Furthermore, Fig. 6D evidences that the number of EDU-positive cells rises after IL-17 A treatment but diminishes with *Piezo1* knockdown, implicating an inhibition of cellular proliferation. Figure 6E reveals that cell migration, significantly promoted by IL-17 A, is reduced following sh-Piezo1 transfection. Contrastingly, Fig. 6F shows that while IL-17 A impedes apoptosis, sh-Piezo1 transfection notably intensifies apoptotic rates. To corroborate the role of PIEZO1 further, HaCaT cells treated with Yoda1, a PIEZO1 agonist, displayed enhanced nuclear localization and activation of p65, as indicated by cell fluorescence in Fig. 7A. Dual staining of PIEZO1 and p65, depicted in Fig. 7B, showed that Yoda1 markedly augmented PIEZO1 expression and increased p65 fluorescence intensity compared to the control. This substantiates the notion that PIEZO1 activates p65. The beneficial effects of PIEZO1 silencing in psoriasis are attributed to the inhibition of the NF-κB signaling pathway.

### PIEZO1 facilitates Keratinocyte-Mediated CD4 + T cell differentiation

**Fig. 8:**
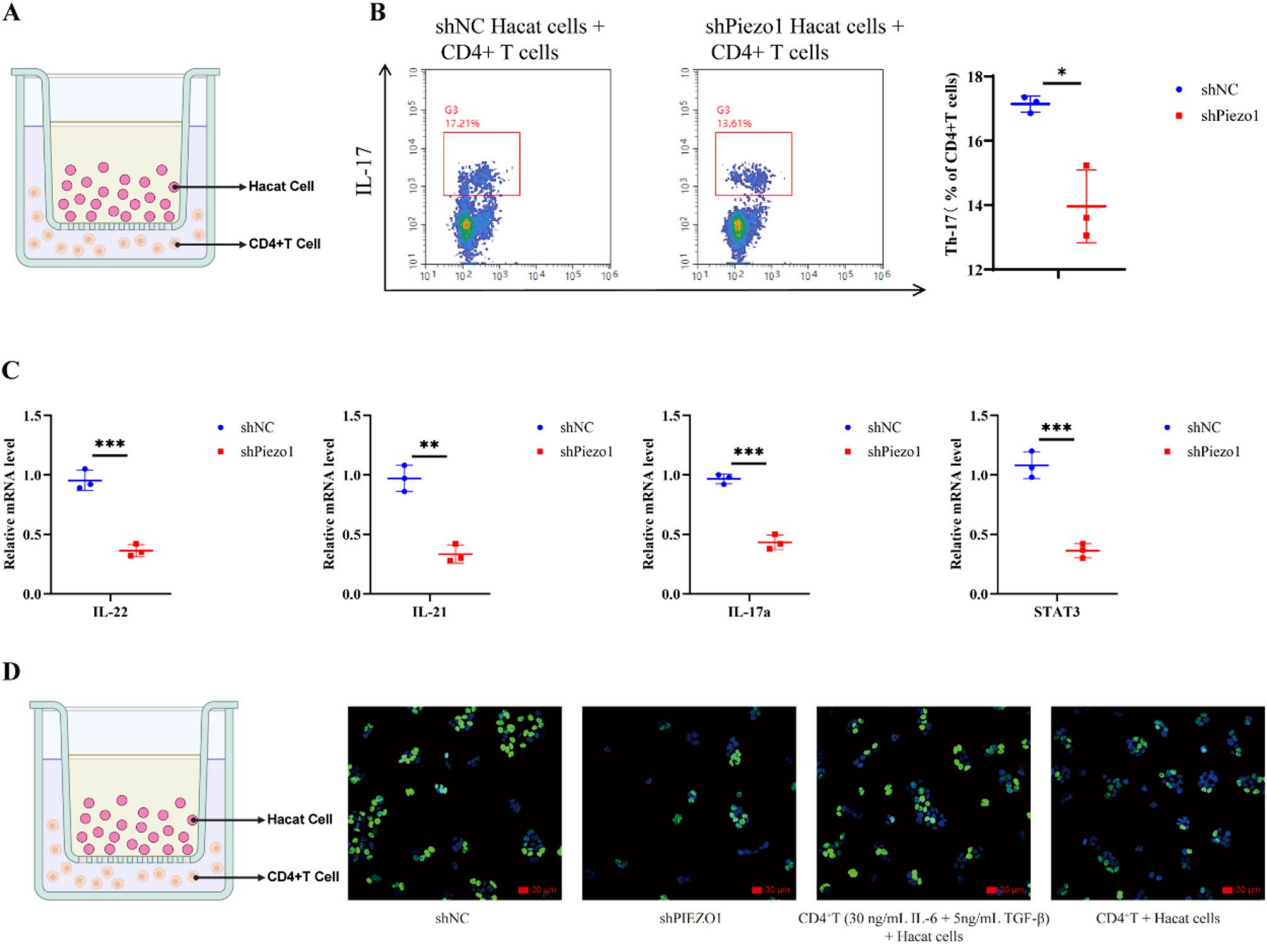
*PIEZO1* p romotes the differentiation of CD4 + T cells mediated by keratinocytes in HaCaT cells transfected with shPiezo1 or shNC. **A **Schematic representation of co-culture **B **The percentage of IL-17 detected in co-culture experiments was quantified and statistical analysis graph was generated from flow analysis data for further evaluation. **C **mRNA expression levels of IL-22, IL-21, IL-17 A, and STAT3 were determined using qPCR to assess their involvement. **D **Schematic representation of co-culture and Cell proliferation ability was assessed across different experimental groups using EdU analysis. *P<0.05, **P<0.01, ***P<0.001,**** *P*<0.0001

**Fig. 9:**
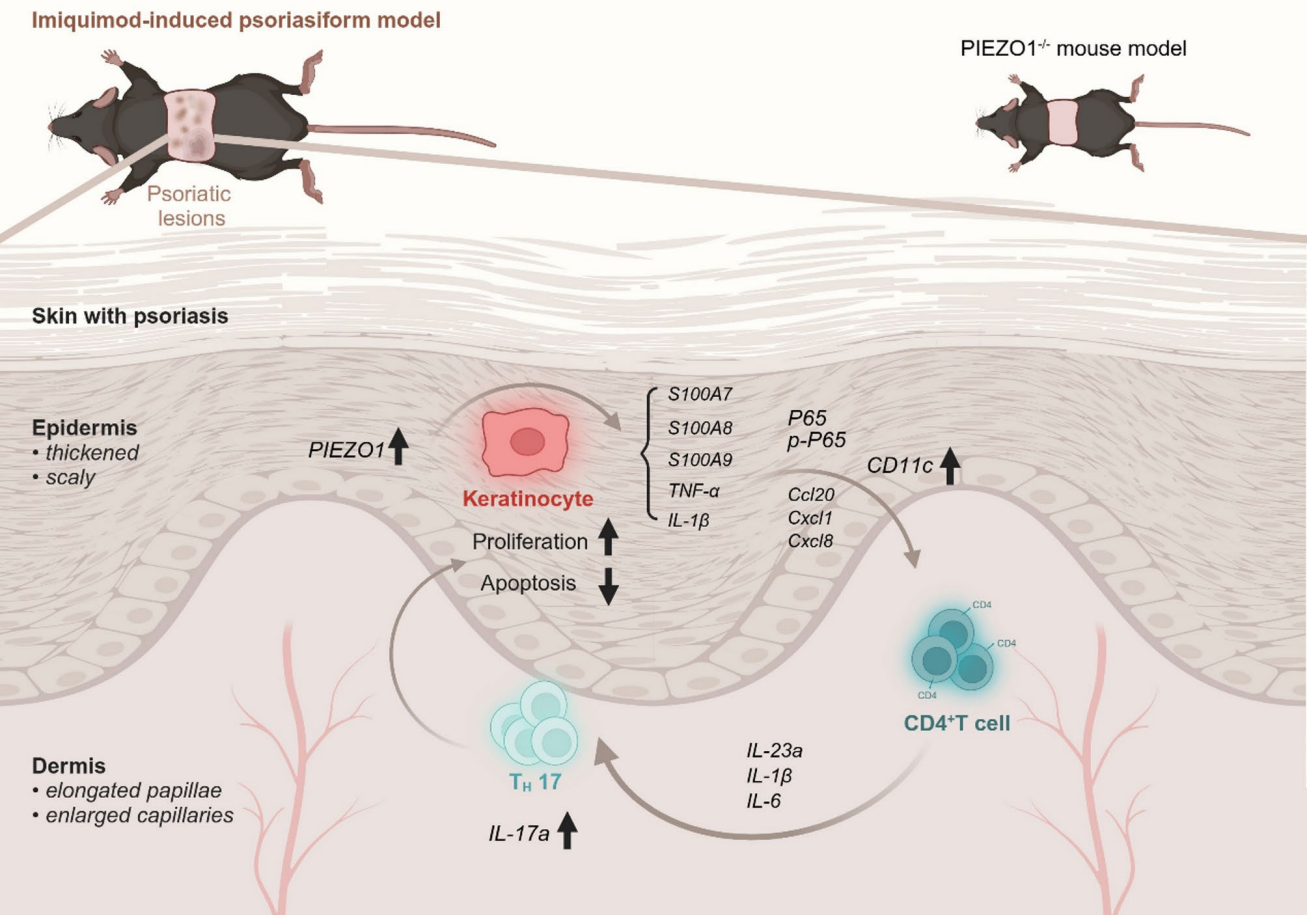
The overexpression of *PIEZO1* is implicated in the molecular mechanisms underlying the pathogenesis of psoriasis

This study investigated the role of keratinocytes in directing the differentiation of Th cells. CD4 + T cells were isolated from mouse splenic lymphocytes and subsequently co-cultured with HaCaT cells (as shown in Fig. 8A), which were subjected to knockdown with sh-NC or shPiezo1. We then performed flow cytometry on CD4 + T cells, which demonstrated that PIEZO1 knockdown in HaCaT cells markedly reduced the proportion of Th17 cells (as shown in Fig. 8B). Moreover, as delineated in Fig. 8C, diminished expression of PIEZO1 was associated with lower mRNA levels of IL-22, IL-21, IL-17a, and STAT3. Additionally, Fig. 8D illustrates that shPIEZO1 transfection into HaCaT cells significantly curtailed the percentage of EdU positive cells. Conversely, administering IL-6 + TGF-β to CD4 + T cells led to an increased fraction of EdU positive cells. Collectively, these findings suggest that PIEZO1 plays a pivotal role in keratinocyte-mediated CD4 + T cell differentiation (Fig. 9).

## Discussion

Psoriasis is a multifactorial chronic inflammatory skin disorder characterized by disrupted skin barrier function, aberrant proliferation of keratinocytes, and dysregulated immune response (Yamanaka et al. 2021). The intricate interplay among keratinocytes (Ni and Lai 2020), dendritic cells, and T cells orchestrates the disease’s characteristic inflammatory milieu dominated by cytokines such as TNF-α, IL-23, and IL-17 (Zhou et al. 2022; Aslani et al. 2023). Recent advancements in understanding the molecular participants involved in psoriasis pathogenesis have provided novel insights and proposed alternative therapeutic targets. The Th17 axis, which plays a crucial role in psoriasis development, has been targeted by emerging therapies (Sharma et al. 2022; Bugaut and Aractingi 2021); however, unraveling the underlying mechanisms of this disease remains an ongoing pursuit in dermatological research.

One such molecule is PIEZO1, a mechanically activated ion channel implicated in various cellular functions, including cell proliferation, differentiation, and migration (Ranade et al. 2014). PIEZO1 has emerged as a cytokine with potential regulatory effects on the Th17 response (Wang et al. 2022; Jairaman et al. 2021); however, its role in psoriatic pathology has been unclear. Our study delves into the intricate biological mechanisms underlying psoriasis, highlighting the prominent role of PIEZO1—a mechanosensitive ion channel—in the etiology of this disease. The results of our study support previous findings that demonstrate increased expression of PIEZO1 in the epidermis of individuals with psoriasis, consistent with numerous recent investigations examining PIEZO1 in other inflammatory conditions. For instance, research have demonstrated an upregulation of PIEZO1 activity in myeloid cells during colitis (Leng et al. 2022), indicating its extensive involvement in the regulation of inflammation. Furthermore, our study observed an attenuated psoriasiform phenotype in mice lacking functional PIEZO1 gene expression; these mice demonstrated a reduction in inflammatory infiltrate and keratinocyte hyperproliferation, consistent with previous studies reporting attenuated inflammation upon inhibition of PIEZO1 activity or expression. For instance, specific deletion of mouse Piezo1 in the intestinal epithelium significantly disrupted gut peristalsis, impeded experimental colitis, and suppressed serum 5-HT levels (Sugisawa et al. 2020). Current research on psoriasis inflammation highlights the Th17/IL-17 axis as a crucial factor (Chen et al. 2022). Our data supports this notion by demonstrating that PIEZO1 upregulation occurs in response to pro-inflammatory cytokines, including IL-17 A. Notably, knockdown of PIEZO1 in HaCaT keratinocyte cell lines resulted in reduced inflammatory response to IL-17 A, confirming its role as an amplifier in the vicious cycle of psoriasis inflammation. These findings suggest that targeting PIEZO1 therapeutically may break this cycle and provide promising insights for future drug development.

Nonetheless, the distinctive transcriptome signature arising from PIEZO1 deficiency in the skin implies a context-specific modulation of inflammation mediated by PIEZO1. In contrast to the mechanisms reported by Yu et al., where PIEZO1 expression was induced through inflammatory cytokine production in astrocytes (Yu et al. 2023), our findings highlight that PIEZO1 activation specifically exacerbates inflammation in keratinocytes. These discrepancies highlight the tissue-specific effects of PIEZO1 and emphasize the complexity of its regulatory role. Our observations regarding mice deficient in PIEZO1 reveal a less severe psoriatic phenotype, aligning with the proposed anti-inflammatory benefits associated with reducing PIEZO1 expression as demonstrated in other inflammatory models (Liu et al. 2024). This outcome is particularly significant in terms of translational potential; it prompts further exploration within the scientific community into utilizing PIEZO1 antagonists or modulators as potential therapeutic agents for psoriasis. The complexity underscores the need for research on cell-specific functions of PIEZO1 and highlights potential pitfalls associated with global targeting.

Furthermore, our transcriptomic analysis highlights the comprehensive impact of PIEZO1 on the expression of inflammatory cytokines and migration-inducing chemokines, providing insights into its involvement in NF-kB pathway engagement. This reinforces the well-established significance of the pathway in psoriasis pathogenesis (Zhu et al. 2024) and reveals a novel role for PIEZO1 as a contributor within this signaling cascade. The interplay between PIEZO1 and the NF-kB/IL-17 pathway depicted in this study expands upon existing knowledge, elucidating how PIEZO1 may modulate the inflammatory state in psoriasis. Previous work by Berenice et al. demonstrated an upregulation of IL-17 and related cytokines in psoriatic lesions (Fischer et al. 2023), which is consistent with our findings but also adds a new layer of interaction through PIEZO1 influence. Inhibiting PIEZO1 appears to attenuate NF-kB signaling, thereby curtailing the production of inflammatory cytokines and chemokines involved in perpetuating psoriatic inflammation. These findings warrant consideration for clinical translation as they identify a multifaceted component that, when modulated, impacts various inflammatory aspects underlying psoriatic pathogenesis.

Despite our intriguing results, the research is not without limitations. Primarily, we are constrained by the use of model systems—mice do not perfectly represent humans, and IMQ-induced psoriasiform dermatitis does not fully replicate psoriasis. This raises concerns about the applicability of mouse data to human pathology. Additionally, it remains uncertain whether PIEZO1 regulation in a human biological context accurately reflects our findings in mice. Furthermore, while our in vitro assays provide valuable insights, they cannot fully capture the complexity of the tissue environment in vivo where multiple signaling pathways and cell interactions occur simultaneously. Addressing these concerns is crucial for advancing PIEZO1-targeted therapies; it requires careful examination of PIEZO1’s role across different skin-cell subtypes and confirmation that targeting this channel does not lead to unforeseen consequences elsewhere. Bridging this gap could be achieved through utilizing patient-derived keratinocytes and immune cells, potentially differentiated from primary human induced pluripotent stem cells (iPSCs), as well as evaluating PIEZO1 inhibitors using more sophisticated human in vitro skin models such as organotypic cultures.

## Conclusion

Our findings reveal the significant role of PIEZO1 in promoting keratinocyte dysfunction, inflammation, and T cell-mediated immunopathology in psoriasis. Our research suggests that PIEZO1 is not only a crucial component for maintaining keratinocyte homeostasis but also a promising therapeutic target for treating psoriasis. Targeting PIEZO1 may offer a novel approach to alleviate inflammation, keratinocyte proliferation, and the abnormal immune response characteristic of psoriatic disease. Consequently, these results may pave the way for developing strategies based on PIEZO1 to advance the management of psoriasis and reduce the burden of this chronic inflammatory skin disorder.

## Data Availability

All data are provided in this study, and the original data can be obtained from the corresponding author upon reasonable request.
